# TiO_2_ Sol-Gel Coated PAN/O-MMT Multi-Functional Composite Nanofibrous Membrane Used as the Support for Laccase Immobilization: Synergistic Effect between the Membrane Support and Enzyme for Dye Degradation

**DOI:** 10.3390/polym12010139

**Published:** 2020-01-06

**Authors:** Qingqing Wang, Tingting Wang, Zihao Lv, Mengting Cui, Ziqiang Zhao, Xiuming Cao, Qufu Wei

**Affiliations:** 1 Key Laboratory of Eco-Textiles, Ministry of Education, Jiangnan University, Wuxi 214122, China; wa_titing@163.com (T.W.); lzh17315534332@163.com (Z.L.); cui1999089@163.com (M.C.); zhao_zi_qiang@163.com (Z.Z.);; 2 Jiangsu Sunshine Group Co., Ltd., Jiangyin 214400, China; caoxium@163.com

**Keywords:** laccase, titanium dioxide, immobilization, nanofiber, electrospinning, dye degradation

## Abstract

Removal of a triphenylmethane dye (crystal violet, CV) by a simultaneous enzymatic-photocatalytic-adsorption treatment was investigated in this work. A desirable synergistic effect on dye treatment was achieved by decorating laccase (Lac) onto the surface of TiO_2_ sol-gel coated polyacrylonitrile/organically modified montmorillonite (PAN/O-MMT) nanofibers prepared by electrospinning. The assembly of Lac on the surface of PAN/O-MMT/TiO_2_ nanofibers was confirmed by confocal laser scanning microscope (CLSM). In comparison with free Lac, the immobilized Lac showed better pH, temperature and operational stabilities, reaching highest relative activity at an optimum pH of 3 and optimum temperature of 50 °C. Therefore, the immobilized Lac displayed a higher degradation efficiency of CV at an initial dye concentration of 100 mg/L, an optimum pH of 4.5 and temperature at 60 °C. Under UV illumination, the CV removal efficiency was further improved by ~20%. These results demonstrated that the Lac-immobilized PAN/O-MMT/TiO_2_ composite nanofibers with a combined effect between the immobilized enzyme and the polymeric support have potential for industrial dye degradation.

## 1. Introduction

Wastewater pollution has been an increasingly serious problem that garners significant attention from society and scientific researchers over the past five decades. Great quantities of wastewater are released from paper, painting, leather, and cosmetics industries, especially the textile industry [1,2]. It is reported that over 10,000 different toxic chemicals such as dyes and dyeing auxiliaries are released from textile dyeing and finishing [3]. Crystal violet (CV) is a triphenylmethane dye providing a deep purple color for dyeing. It is a calcitrant and hazardous dye that can persist in the environment for a long time, affect cell division process of living organisms and promote tumor growth in marine life [4,5,6]. Therefore, it becomes important for dyes in wastewater (e.g., crystal violet) to be degraded before the final discharge [7]. In terms of treatments for textile wastewater, traditional methods such as physical, chemical, electrochemical methods are costly and could produce secondary pollutants [8,9,10]. In contrast, biodegradation attracts more interest from researchers owing to its low cost, excellent efficiency and environmental friendliness [11,12].

Laccase (Lac, benzenediol: oxygen oxidoreductase, EC 1.10.3.2) is a multi-copper oxidase that can catalyze the degradation of a wide variety of substrates while avoiding the co-formation of toxic by-products [13,14]. However, laccase is sensitive to industrial operating conditions, so the reaction time should be significantly reduced to increase the laccase utility. To optimize the degradation, titanium dioxide (TiO_2_) is being combined with Lac due to its desirable photocatalytic capability in the removal of textile dyes. Aura et al. [15] proposed a two-step method firstly using *Trametes versicolor* for biodegradation, followed by photocatalytic degradation employing UV/TiO_2_/Ru_x_Se_y_ to treat wastewater from papermaking, resulting in a 92% color removal and 99% chlorophenol degradation after about 96 h. Luisa et al. [9] also studied the removal of chlorophenol while using TiO_2_/UV photocatalysis first and then *Trametes pubescens*, ultimately achieving a degradation rate of 100%. These works show that the sequence of photocatalytic and biocatalytic processes has no influence on the final treatment efficiency. Therefore, it is promising for Lac and TiO_2_ to work simultaneously, thus further improving the dye degradation efficiency and reducing energy consumption.

Jia et al. [16] studied the co-action system of TiO_2_ and immobilized Lac on the degradation of 2,4-dichlorophenol. Compared with free Lac or TiO_2_/UV, the degradation efficiency of simultaneous photocatalytic-enzymatic treatment was significantly increased, demonstrating a synergy between the TiO_2_/UV and immobilized Lac. To further improve the overall dye removal capacity, Lac was immobilized on TiO_2_ particles through covalent bonding methods [17,18,19]. However, the Lac conjugated TiO_2_ particles showed low stability, reusability and recyclability. To circumvent this limitation, various attempts were made for the immobilization of Lac and TiO_2_.

A number of scaffolds have been utilized in enzyme and TiO_2_ immobilization, including cellulose nanofibers [20,21], ceramic membrane [22], graphene oxide membrane [23], silica nanoparticles [24], magnetic nanoparticles [25,26,27] and artificial polymers [28,29,30]. Among the carriers of Lac and TiO_2_, electrospun nanofibrous membranes have been proven to have great potential in wastewater treatment, especially with the addition of adsorbent material in the process of electrospinning [31]. Electrospinning is a globally recognized practical technology for the fabrication of polymeric membranes due to its ease-of-operation, low cost, excellent adjustability and malleability [32,33]. Electrospun polymer is efficient for absorbing and filteringcontaminants [34,35], which can be attributed to its porous structure, high specific surface area, electrostatic attraction, nonpolar attraction, intermolecular force, and functional groups [36]. In our previous work, the addition of adsorbent montmorillonite (MMT) has been testified to increase the adsorption ability of the polymeric membrane [37].

**Figure 1:**
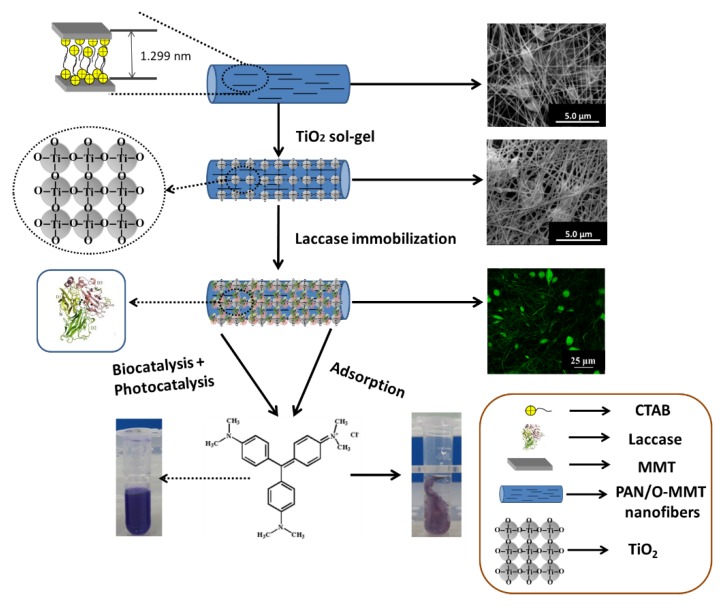
Schematic illustration of polyacrylonitrile/organically modified montmorillonite (PAN/O-MMT) composite nanofibers functionalized by sol-gel coating of TiO_2_ and then used as support for laccase and its application in crystal violet (CV) treatment.

Herein, we proposed electrospun PAN/organically modified montmorillonite (O-MMT) nanofibrous membrane, coated by TiO_2_ sol-gel before the immobilization of laccase (Lac) to prepare a functional composite nanofibrous material, innovatively combining the adsorption abilities of O-MMT and electrospun nanofibrous membrane, enzymatic degradability of laccase as well as photo-catalytic degradability of TiO_2_ (Figure 1). In addition to physical characterization using SEM and confocal laser scanning microscopy (CLSM), the kinetic, optimum pH and temperature of both free and immobilized Lac were studied in detail, and their thermal and operational stabilities were also evaluated. The effect of initial dye concentration, optimum pH and temperature on degradation of crystal violet (CV) was also investigated. In addition, the removal efficiency with and without the presence of UV light was compared to study the synergistic effect of simultaneous enzymatic-adsorption or enzymatic-photocatalytic-adsorption treatment. To this end, our findings demonstrate that TiO_2_ sol-gel coated PAN/O-MMT nanofibers is an excellent support for laccase immobilization and the Lac-immobilized PAN/O-MMT/TiO_2_ functional composite nanofibrous membrane is a promising material with application potential for dye degradation.

## 2. Materials and Methods

### 2.1. Materials

Polyacrylonitrile (PAN) (*M*w = 50,000 g/mol) powder was purchased from Shangyu Wu & Yue Economic and Trade Co., Ltd. (Zhejiang, China). Organically modified montmorillonite (O-MMT, modified by CTAB) was obtained from Zhejiang Fenghong New Material Co., Ltd. (Zhejiang, China). Laccase and 2,2′-Azino-bis(3-ethylbenzothiazoline-6-sulfonic acid) diammonium salt (ABTS) were supplied by Sigma–Aldrich, China. Crystal violet (CV), *N*,*N*-dimethyl formamide (DMF), sodium acetate and acetic acid were all purchased from Sinopharm Chemical Reagent Co., China. All reagents and solvents were used as received.

### 2.2. Preparation of Lac-Immobilized PAN/O-MMT/TiO_2_ Nanofibers

The pre-cursor solution was prepared as follows: first, O-MMT and PAN solid powder were dried at 50 °C for 12 h before use. Then, 0.15 g of O-MMT powder was fully dissolved in 20 mL of DMF solution after sonication for 3 h. Then, 3 g PAN powder was added into the solution before magnetic stirring for 24 h. The PAN/O-MMT nanofibers were electrospun in an apparatus employing a high voltage (15 kV) power supply. The flow rate was set as 0.5 mL/h and the constant distance between needle tip and collector was 15 cm. Finally, it was collected on a roller covered with aluminum foil.

The PAN/O-MMT/TiO_2_ nanofibers was prepared as follows: first, Ti(OC_4_H_9_)_4_ was added into 6 mol/L HCl dropwise and stirred for 30 min to prepare the TiO_2_ sol. Then, it was added into deionized water until the ratio of Ti and HCl became 0.83:1. Sequentially, the solution became homogeneous after magnetically stirring for 1 h [38]. After 5 days aging at room temperature, the PAN/O-MMT nanofibers was immersed into the TiO_2_ sol for 12 h. Finally, the as-prepared PAN/O-MMT/TiO_2_ membrane was dried at 60 °C for 10 min before further use.

Then, the PAN/O-MMT/TiO_2_ nanofibers were functionalized after the immobilization of Lac. A 400 mg of PAN/O-MMT/TiO_2_ nanofibrous membrane was immersed into a 250 mL flask containing 100 mL of 0.3 g/L Lac in sodium acetate solution (pH = 4) and adsorbed by electro-static force between ionized enzyme and cationic TiO_2_ precursors. After shaking at 25 °C for 3 h, the membrane was thoroughly washed by buffer solution. Later, the Lac-immobilized PAN/O-MMT/TiO_2_ nanofibrous membrane was stored at 4 °C.

### 2.3. Morphology

To observe the morphology of PAN/O-MMT before and after TiO_2_ coating, scanning electron microscopy (SEM, SU1510) was performed at an accelerating voltage of 12.5 kV with a magnification of 10 k.

To verify Lac has been successfully immobilized on the PAN/O-MMT/TiO_2_ nanofibrous membrane, fluorescein isothiocyanate (FITC)-labeled Lac was applied. After immobilization, the FITC solution was thoroughly washed by buffer solution. Finally, confocal laser scanning microscope (CLSM, TCS SP8) was employed for visualization of Lac at an excitation wavelength of 488 nm.

### 2.4. Laccase Activity and Stability Assays

The activities of both free and immobilized Lac were determined by oxidizing ABTS in sodium acetate buffer solution at 30 °C [39]. Next, 0.1 mL of enzyme solution or Lac-immobilized membrane (2 cm × 2 cm) was added into 2.9 mL ABTS (15 mM) solution for 3 min, then the absorbance of the solution was recorded at the wavelength of 420 nm by UV-1700 spectrophotometry. All assays were performed in triplicate. Experimental data were reported as mean ± standard deviation.

To study the kinetics, ABTS solution (pH = 4) in the range of 0.1–1 mM was oxidized by free and immobilized Lac at 30 °C. The kinetic parameters (*K_m_* and *V_max_*) were calculated according to Michaelis–Menten equation. Here, *K_m_* is the Michaelis constant, representing the concentration of enzyme when the reaction rate reaches half of *V_max_* (the maximum reaction rate).

The optimum pH was assayed by adding free and immobilized Lac into buffer solutions at different pH varying from 2 to 6 at 30 °C for 10 min. To determine the optimum temperature, the enzymes were respectively added into buffer solutions (pH = 4) at different temperatures ranging from 20–70 °C for 10 min before incubation at 30 °C for 5 min. Then, all the samples were used to react with ABTS for 3 min to test the relative activities of Lac under different conditions.

For thermal stability studies, the relative activities of free and immobilized Lac were studied at 20 °C, 30 °C, 40 °C and 50 °C and recorded every 2 h. The operational stability at 30 °C and 50 °C was evaluated by testing the activity of Lac after each repeated utilization (8×). During each interval, the membrane was recovered and sufficiently washed with buffer solution.

### 2.5. Degradation of CV

To determine the amounts (0, 3, 5, 10 and 20 μL) of ABTS mediator (15 mM), 0.1 mL of 0.3 g/L free Lac was employed to degrade 40 mL of 50 mg/L CV solution (pH = 4) at 40 °C for 5 h and concentration was recorded every hour at a wavelength of 590 nm by employing a UV-1200 spectrophotometer. All assays were performed in triplicate. Experimental data were reported as mean ± standard deviation.

Different concentrations (10, 25, 50, 100 and 150 mg/L) of 40 mL CV solution (pH = 4) were degraded by 100 mg of immobilized Lac at 40 °C to study the effect of its initial concentrations on removal efficiency. To investigate the effect of pH on CV degradation, 100 mg of immobilized Lac was used to remove 40 mL of 50 mg/L CV solution in the pH range of 2–6 at 40 °C. The effect of temperature on CV removal efficiency was also studied by employing 100 mg of immobilized Lac to remove 40 mL of 50 mg/L CV solution (pH = 4) at 20–70 °C. The CV removal efficiency of each group was recorded after 5 h.

To assess the influence of UV light on laccase activity, free Lac was employed to degrade 40 mL of 50 mg/L CV solution (pH = 4) at 40 °C. To evaluate the CV removal efficiency under UV illumination, immobilized Lac was used to degrade 40 mL of 50 mg/L CV solution (pH = 4) at 40 °C. The CV removal efficiency of each group was recorded at different intervals.

## 3. Results and Discussion

### 3.1. Morphology

**Figure 2:**
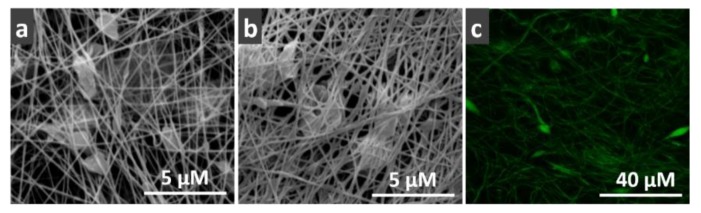
SEM images of (**a**) PAN/O-MMT, and (**b**) PAN/O-MMT/TiO_2_ nanofibers. (**c**) Confocal laser scanning microscope (CLSM) micrograph of Lac-immobilized PAN/O-MMT/TiO_2_ nanofibers.

The morphology of the PAN/O-MMT nanofibers before and after TiO_2_ coating was compared using SEM (Figure 2a,b). It can be seen from Figure 2a that PAN/O-MMT electrospun nanofibers had a beaded structure with very fine, randomly distributed nanofibers (138.27 ± 23.22 nm). After TiO_2_ coating, the nanofibers became more adhesive to each other while the other features were well maintained. The PAN/O-MMT/TiO_2_ nanofibers coated with electrostatically adsorbed Lac can be observed in the CLSM image (Figure 2c). The FITC-labeled Lac was uniformly and continuously distributed throughout the three-dimensional nanofiber network, confirming a good compatibility between the enzyme molecules and the solid support.

### 3.2. Enzymatic Activity

Table 1 shows the Lac loading and retention activity of immobilized Lac as well as the kinetic parameters of both free and immobilized Lac. The Lac loaded on PAN/O-MMT/TiO_2_ was 342 mg per gram of the membrane, which is higher than our previous work using PAN/O-MMT nanofibrous membrane with the covalent immobilization method [40]. The retention activity of the immobilized Lac in comparation with free Lac was 82.5%, which is also much higher than the previous work (73.5% of its specific activity).

After immobilization, the *K_m_* value was inevitably increased while the *V_max_* decreased to some extent, which could be attributed to the limited diffusion ability of the immobilized Lac on the scaffold. Therefore, the accessibility of the immobilized Lac and substrate decreased, resulting in a lower enzyme activity [30].

### 3.3. Optimum pH and Temperature

In addition to the nature of enzyme and the binding interaction between enzyme and scaffold, operating conditions also affect the enzymatic activity. Investigation of optimum pH and temperature gives information on suitable conditions to achieve relatively higher enzymatic activity, which is vital for its further applications. Enzyme activity was detected by catalyzing the oxidation of ABTS under different pHs and temperatures. The highest enzyme activity at a specific pH or temperature was normalized as 100% and the relative activities at other conditions were calculated accordingly.

**Figure 3:**
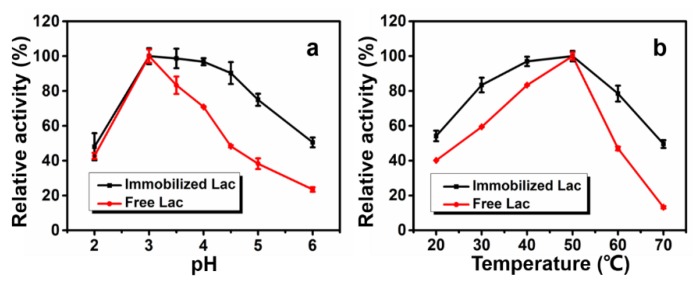
Effects of pH (**a**) and temperature (**b**) on Lac activity.

The effect of pH on laccase activity before and after immobilization is shown in Figure 3a, immobilized Lac had much higher relative activity retention than free Lac, indicating that the free Lac was more sensitive to the fluctuation of pH. In addition, the optimum pH of both free and immobilized enzyme was pH = 3. This suggests that the charge density of PAN nanofibers and the coated TiO_2_ sol on its surface was not sufficient to change the microenvironment of the enzyme, which is consistent with other reported research [41].

However, the relative activity of immobilized Lac on PAN/O-MMT/TiO_2_ nanofibrous membrane between pH range of 3–6 was higher than free Lac. This was mainly because that the MMT intercalated within PAN can adsorb H^+^ in the buffer, making the microenvironment around the immobilized laccase more acidic. However, the immobilized Lac on PAN/O-MMT/TiO_2_ nanofibrous membrane maintained the same optimum pH as free Lac, because a large amount of cations such as TiO_2_^+^/Ti(OH)_2_^2+^ generated during hydrolysis, distributed onto the PAN/O-MMT/TiO_2_ nanofibrous membrane and competed with the H^+^ in the buffer to prevent the microenvironment around the immobilized Lac being more acidic. Generally, the immobilized Lac demonstrated a higher retention activity over higher pH, broadening the operational pH range.

Increase of temperature can speed up the reaction between the enzyme and the substrate to some extent. However, when the temperature becomes too high, the enzyme would be denatured and loss its activity. Figure 3b shows the relative activity of the free and immobilized Lac over a temperature range from 20 °C to 70 °C. The optimum temperature with the highest relative activity for free and immobilized Lac was found to be located at 50 °C. Generally, the stability of the immobilized Lac was better than that of free Lac in the fluctuation of temperature. The free Lac lost ~60% activity at 20 °C and ~90% at 70 °C, while the immobilized Lac lost ~45% at 20 °C and ~50% at 70 °C. The stability of Lac activity despite fluctuations in temperature was due to the multi-linkages among nanofibers that can attenuate the mobility of enzyme molecular and help Lac maintain a stable conformation [23,40,42].

### 3.4. Thermal Stability and Reusability

**Figure 4:**
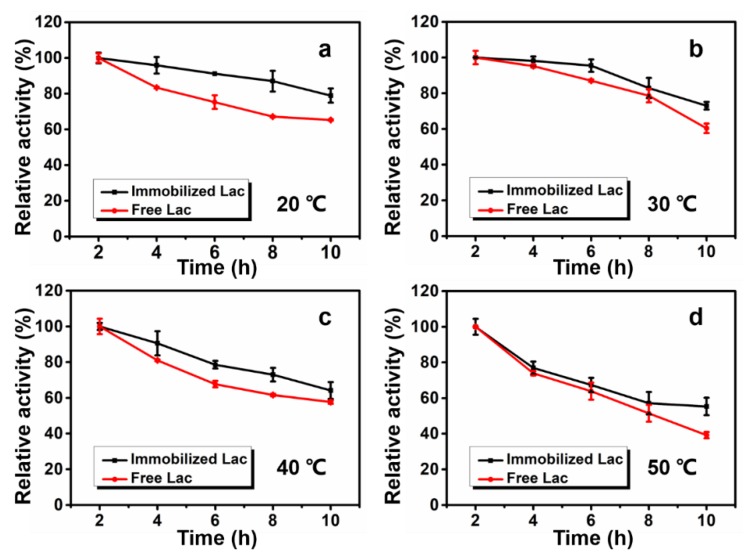
Thermal stability of free and immobilized Lac at: (**a**) 20 °C, (**b**) 30 °C, (**c**) 40 °C and (**d**) 50 °C.

The thermal stability was evaluated ranging from 20 °C to 50 °C and recorded every 2 h, as shown in Figure 4. With the increase of incubation temperature, the thermal stability of both free and immobilized Lac decreased, however, the immobilized Lac displayed better thermal stability than free Lac at different temperatures.

At 20 °C, the immobilized Lac maintained around 85% of its initial activity after 8 h, while the free Lac only retained 67% (Figure 4a). At 30 °C, the enzyme activity of immobilized Lac was not greatly decreased and 82% of its initial activity was maintained after 8 h (Figure 4b). At 40 °C, the immobilized Lac retained above 70% of its initial activity after 8 h (Figure 4c). Even at 50 °C (8 h), it retained approximately 60% of its initial activity (Figure 4d), which is similar to the results of previous research [43]. These results verified a better thermal stability of Lac after covalent immobilization, which can be attributed to the decrease of enzyme flexibility and conformational changes [44].

**Figure 5:**
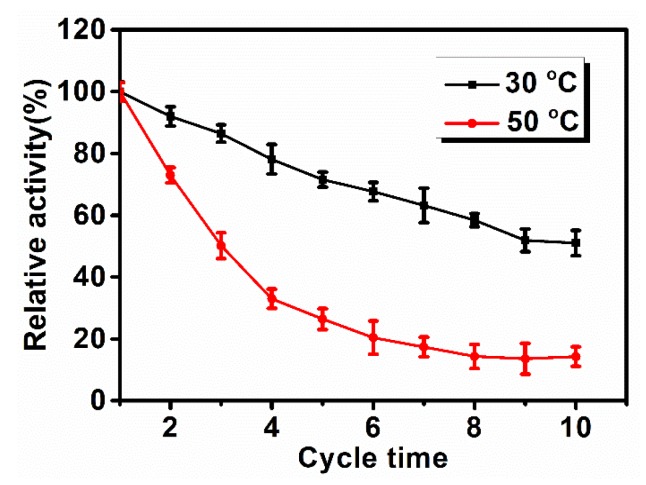
Operational stability of immobilized Lac enzyme at 30 °C and 50 °C.

Reusability is also essential in the study of enzyme immobilization [45]. Figure 5 illustrates that the reusability of the immobilized Lac decreased dramatically when the temperature increased from 30 °C to 50 °C. At 30 °C after 8 cycles, the immobilized Lac retained nearly 60% of its initial activity; while at 50 °C, it dropped below 50% of its initial activity directly after 2 cycles. These results indicate the good reusability of Lac-immobilized PAN/O-MMT/TiO_2_ nanofibrous membrane at a lower temperature and its great potential in dye degradation and biotechnological applications.

### 3.5. Degradation of Crystal Violet (CV)

#### 3.5.1. Effect of ABTS Concentration

**Figure 6:**
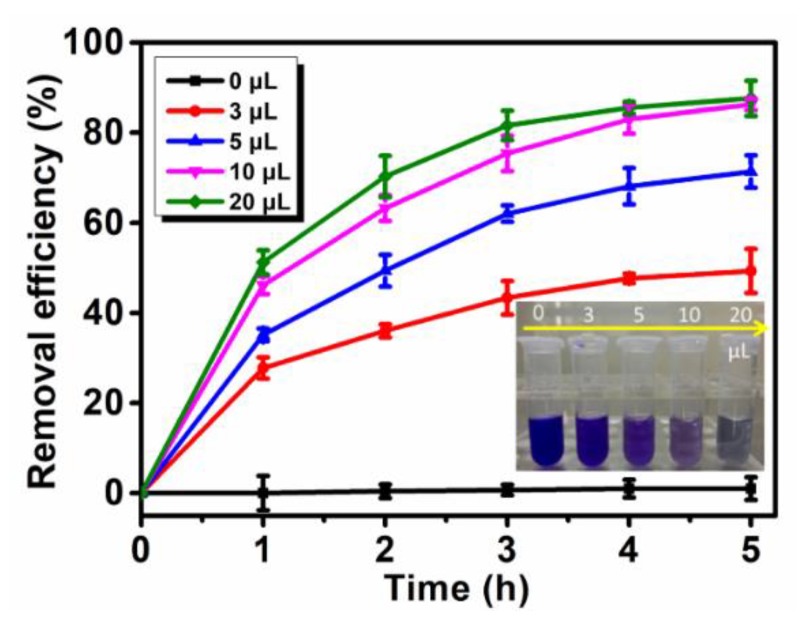
The relationship between the amounts of ABTS (15 mM) added into the system and the dye removal efficiency as well as the inserted photo of crystal violet (CV) (50 mg/L) after removal by free Lac at 40 °C for 5 h.

Investigation of the optimum concentration of the mediator ABTS was carried out by employing free Lac to degrade 50 mg/L CV solution. The removal efficiency of CV mediated by incremental amount of ABTS was shown in Figure 6. It can be observed that Lac itself showed negligible effect on CV degradation, while with the gradual increase of the ABTS addition, the dye removal efficiency greatly improved. As shown in the photo, the dye solution was almost discolored by free Lac when 20 μL of ABTS was added. So, 20 μL is determined as the appropriate dosage.

#### 3.5.2. Effect of Initial Dye Concentration

**Figure 7:**
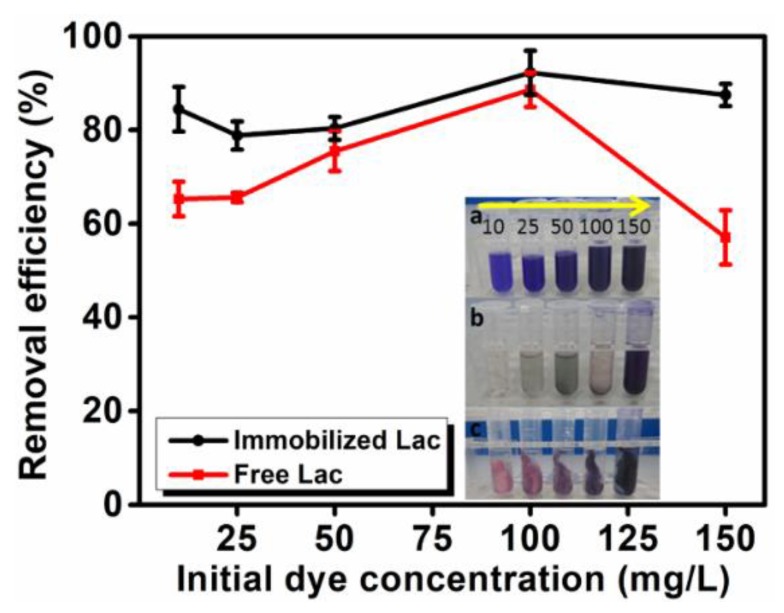
Effect of initial dye concentration on CV removal efficiency. Digital photograph of (**a**) initial CV (from left to right are 10, 25, 50, 100, 150 mg/L, respectively); the corresponding CV solutions after removal by (**b**) free Lac and (**c**) immobilized Lac at 40 °C for 5 h.

Figure 7 shows the effect of different initial CV concentrations on the dye removal efficiency by free and immobilized Lac. It can be observed that the immobilized Lac showed an overall higher and more stable dye removal efficiency than that of free Lac in the dye concentration span from 10 mg/L to 150 mg/L.

Along with the increased initial CV concentration from 10 mg/L to 150 mg/L, the removal efficiency by free and immobilized Lac fluctuated with a similar trend. When the CV was degraded by free Lac, suspension of insoluble particles was generated when the initial dye concentration was 25 mg/L and they started to agglomerate and precipitate when the concentration was further raised (Figure 7b). For the immobilized enzyme, the phenomenon of its dye removal process was different from that of free enzyme. When the concentration was 25 mg/L, immobilized Lac showed a slightly decreased CV removal ratio from aqueous solution compared with that of 10 mg/L dye solution, which is attributable to the fact that CV was not easily adsorbed by the imbedded MMT due to the diffusional limitations at low concentration [46]. When the initial concentration increased to 100 mg/L, both the free and immobilized Lac displayed good CV removal efficiency. The immobilized enzyme membrane achieved highest removal efficiency of 92% while degrading 100 mg/L CV. However, when the dye concentration further increased from 100 mg/L to 150 mg/L which is beyond the degradation capacity of the material, the removal efficiency of free Lac was sharply decreased while the immobilized Lac showed a negligible decreasing trend. Furthermore, unlike that of the free laccase, no secondary pollution was generated and a clear aqueous solution was observed after the treatment using immobilized Lac.

#### 3.5.3. Effect of pH and Temperature

**Figure 8:**
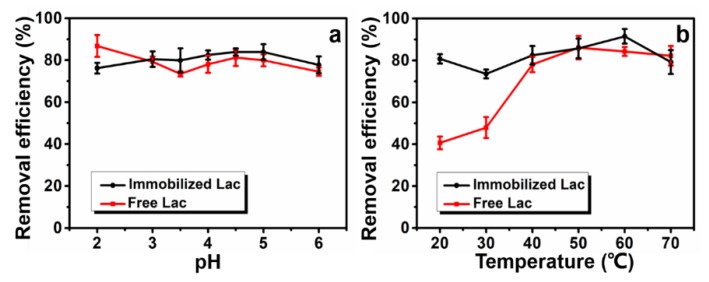
Effect of pH value (**a**) and temperature (**b**) on CV (50 mg/L) removal efficiency.

The removal efficiency of CV can be also affected by variation of pH and temperature, the results are shown in Figure 8. Generally, immobilized Lac showed more stable removal efficiency than free Lac in the pH range of 2–6 (Figure 8a). When the pH was low, large quantities of H^+^ and TiO^2+^/Ti(OH)_2_^2+^ in solution competed with dye molecules for adsorption sites on O-MMT so that the removal efficiency of immobilized Lac was relatively lower [41]. Along with the increasing of pH from 2 to 4.5, the removal efficiency of CV slightly increased due to the enhanced adsorption capacity of O-MMT to CV in a less acidic environment. However, the Lac showed lower activity when the buffer solution become more alkaline (see Figure 3a), thus reducing the removal efficiency. In short, the immobilized Lac reached maximum efficiency at pH 4.5, where adsorption and catalysis capacity achieved the best equilibrium.

The effect of temperature (20–70 °C) on CV degradation is shown in Figure 8b. It is already known that the optimum temperature with the highest Lac activity located at 50 °C for both free and immobilized Lac (Figure 3b). Not surprisingly, free Lac reached the best relative activity at its optimum temperature (50 °C). In terms of immobilized Lac, diffusion of CV molecules can be enhanced under higher temperature, therefore, the adsorption activity of fiber matrix was improved. But as the temperature continued to increase, CV removal efficiency decreased due to the partial denaturation of Lac. It can be concluded that the immobilized Lac reached the maximum removal efficiency at 60 °C by the synergistic effects of adsorption and catalysis.

#### 3.5.4. Effect of UV

**Figure 9:**
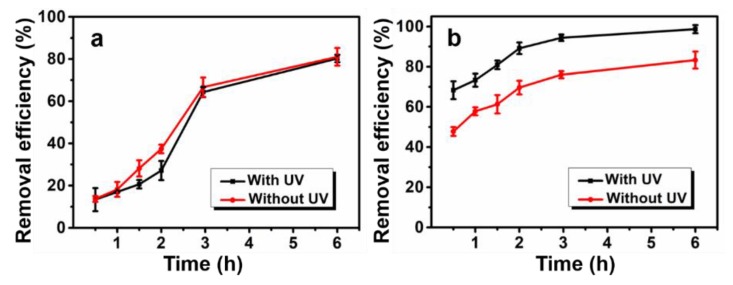
Effect of UV on dye degradation: (**a**) free laccase; (**b**) immobilized Lac.

Figure 9 illustrates CV removal efficiency by free and immobilized enzyme with and without the existence of UV light. As can be seen from Figure 9a, upon UV illumination, free Lac became slightly deactivated and relatively lags behind at the time span of 1–3 h. However, when the enzyme treatment lasted for 6 h, the dye removal efficiency reached the same level for both UV illuminated and non-illuminated system, indicating a minimal effect of the UV light on the Lac activity.

When the dye solution was treated by immobilized Lac, the dye removal efficiency with UV illumination was generally 20% higher than that of the non-illuminated solution over the recorded period, reaching a final dye removal ratio of ~95% (Figure 9b). Therefore, it can be inferred that the introduction of TiO_2_ onto the support can further improve the degradation efficiency through a synergy of photocatalytic and biological effect.

## 4. Conclusions

Electrospun PAN/O-MMT composite nanofibers functionalized by sol-gel coated TiO_2_ were successfully prepared and further used as a matrix to immobilize laccase. It was confirmed by CLSM that laccase was uniformly immobilized onto PAN/O-MMT/TiO_2_ nanofibers at a loading of 342 mg per gram of support. After immobilization, the optimum temperature of Lac remained unchanged at 50 °C, while optimum pH of immobilized Lac changed to higher pH. Compared with free Lac, the pH and thermal stability of the immobilized Lac was improved and the reusability was better retained at lower temperature. Therefore, Lac-immobilized PAN/O-MMT/TiO_2_ nanofibers showed better CV removal capacity, which was hardly influenced by elevated pH and temperature. It reached its highest removal efficiency at an optimal pH of 4.5 and temperature of 60 °C. Under UV illumination, the activity of Lac was hardly affected by UV light. The immobilized Lac combined with photosensitive TiO_2_ improved CV removal efficiency by 20% and reached a final dye removal ratio of ~95% after 6 h illumination, attributed to the synergistic effects of adsorption, biocatalysis and photocatalysis. These results indicate that the Lac-immobilized PAN/O-MMT/TiO_2_ nanofibrous membrane demonstrates enormous potential for removing dyes in industrial wastewaters. It also provides a new approach combining adsorption, biocatalysis and photocatalysis for the elimination of pollutants from environment.

## Figures and Tables

**Table 1:** Enzyme loading, retention activity and kinetic parameters of the free and immobilized laccase (Lac).

Lacs	Enzyme Loading (mg/g Membrane)	Retention Activity (%)	*K_m_* (µmol/mL)	*V_max_* (µmol/mg·min)
Free Lac	-	-	0.12	595.24
Immobilized Lac	342	82.5	0.35	292.71
